# Analysis of necroptotic proteins in failing human hearts

**DOI:** 10.1186/s12967-017-1189-5

**Published:** 2017-04-28

**Authors:** Adrián Szobi, Eva Gonçalvesová, Zoltán Varga, Przemyslaw Leszek, Mariusz Kuśmierczyk, Michal Hulman, Ján Kyselovič, Péter Ferdinandy, Adriana Adameová

**Affiliations:** 1 Department of Pharmacology & Toxicology, Faculty of Pharmacy, Comenius University in Bratislava, Odbojárov 10, 832 32 Bratislava, Slovakia; 2 Department of Heart Failure & Transplantation, The National Institute of Cardiovascular Diseases, Bratislava, Slovakia; 3 Department of Pharmacology & Pharmacotherapy, Semmelweis University, Budapest, Hungary; 4 Institute of Cardiology, Warsaw, Poland; 5 Clinic of Heart Surgery, The National Institute of Cardiovascular Diseases, Bratislava, Slovakia

**Keywords:** Heart failure, Cell death, Necroptosis, MLKL

## Abstract

**Background:**

Cell loss and subsequent deterioration of contractile function are hallmarks of chronic heart failure (HF). While apoptosis has been investigated as a participant in the progression of HF, it is unlikely that it accounts for the total amount of non-functional tissue. In addition, there is evidence for the presence of necrotic cardiomyocytes in HF. Therefore, the objective of this study was to investigate the necroptotic proteins regulating necroptosis, a form of programmed necrosis, and thereby assess its potential role in human end-stage HF.

**Methods:**

Left ventricular samples of healthy controls (C) and patients with end-stage HF due to myocardial infarction (CAD) or dilated cardiomyopathy (DCM) were studied. Immunoblotting for necroptotic and apoptotic markers was performed. Triton X-114 fractionated samples were analyzed to study differences in subcellular localization.

**Results:**

Elevated expression of RIP1 (receptor-interacting protein), pSer^227^-RIP3 and its total levels were observed in HF groups compared to controls. On the other hand, caspase-8 expression, a proapoptotic protease negatively regulating necroptosis, was downregulated suggesting activation of necroptosis signaling. Total mixed-lineage kinase domain-like protein (MLKL) expression did not differ among the groups; however, active cytotoxic forms of MLKL were present in all HF samples while they were expressed at almost undetectable levels in controls. Interestingly, pThr^357^-MLKL unlike pSer^358^-MLKL, was higher in DCM than CAD. In HF, the subcellular localization of both RIP3 and pThr^357^-MLKL was consistent with activation of necroptosis signaling. Expression of main apoptotic markers has not indicated importance of apoptosis.

**Conclusions:**

This is the first evidence showing that human HF of CAD or DCM etiology is positive for markers of necroptosis which may be involved in the development of HF.

**Electronic supplementary material:**

The online version of this article (doi:10.1186/s12967-017-1189-5) contains supplementary material, which is available to authorized users.

## Background

Progressive cell death resulting in functional impairment of the myocardium is a characteristic feature of chronic heart failure (HF). Since regenerative capabilities of the heart are limited the most crucial factor determining cardiac function in this pathological process is the number of viable functional cardiomyocytes [1]. Therefore, understanding which cell death modalities underlie HF-associated cell loss is of a prime importance in order to devise effective pharmacological interventions. Presence of apoptosis, a prominent constituent of the programmed cell death group, in HF has been reported both in clinical and experimental studies [2, 3]. However, the observed percentage of apoptosis positive cells (0.1–0.8%) is likely to be too low to explain the majority of cell death occurring during HF [1, 3, 4]. On the other hand, the relevance of cardiomyocyte necrosis, another major type of cell death, in HF is poorly defined. Of note, some reports have shown that necrotic cardiomyocytes significantly outnumber the apoptotic ones, at least in end-stage HF [1, 5]. Current knowledge about necrosis has been advanced and it has been suggested that necrotic processes are orchestrated by strictly regulated signaling pathways [6]. The most thoroughly described subtype of programmed necrosis is necroptosis which depends on the presence of receptor-interacting protein kinase 1 and 3 (RIP1, RIP3) and mixed-lineage domain-like protein (MLKL) [6]. Exact execution mechanisms of necroptosis are still a matter of investigation; contemporary data implicate that MLKL phosphorylation at Thr^357^/Ser^358^ and oligomerization, secondary to RIP1 and RIP3 activation, leads to membrane permeabilization, ionic dysbalance, oncosis and subsequent cell rupture [7, 8]. Unlike apoptosis, necroptosis is a completely caspase-independent form of cell death; however, caspase-8 (csp-8) is known to indirectly regulate necroptosis activation in a negative manner. In fact, cleavage of RIP1 and RIP3 by caspase-8 prevents necroptotic signaling while simultaneously promoting apoptosis [6]. The importance of necroptosis has been shown mainly in non-cardiac pathologies [9] and recently, it has also been demonstrated in rodent reperfused hearts subjected to previous acute global and regional ischemia [10, 11] as well as in a model of cardiomyopathy [12, 13]. Relevance and proposed mechanisms of necroptotic cell death in heart failure have been reviewed elsewhere [14]. However, there is essentially no experimental evidence about its role in failing hearts irrespective of origin. Therefore, here we analyzed the expression of RIP1, pSer^227^-RIP3, RIP3 and cytotoxic phosphorylated forms of MLKL (pThr^357^-MLKL and pSer^358^-MLKL) in human myocardium from end-stage HF patients due to cardiomyopathy post myocardial infarction (CAD) and dilated cardiomyopathy (DCM). The extent of necroptosis in these types of HF was compared to that of healthy control hearts. In addition, to provide a more complex picture of the signaling environment for necroptosis, we looked at the main negative regulator of necroptosis, csp-8, as well as certain apoptotic proteins.

## Methods

### Human samples

Samples of left ventricles (LVs) from failing hearts were obtained from hearts explanted during cardiac transplantation. Care was taken to ensure to avoid fibrotic, vascular and adipose tissue. Right after excision the samples were rinsed, blotted to dryness and snap-frozen in liquid nitrogen. Excised samples were kept at −80 °C for long-term storage. In total 6 samples of failing hearts of ischemic etiology, 10 samples of failing hearts due to and 4 control samples (C) were employed in the study. All HF patients were of New York Heart Association (NYHA) class III–IV. Control samples were obtained from healthy donors aged 22–44 years, whose hearts could not be used for transplantation due to medical or technical reasons. They received dopamine (1.5–3 µg/kg/min) as well as fluids/colloids before harvest and presented preserved systolic/diastolic function in echocardiographic examination. Patient characteristics (Additional file 1: Table S1) are described in the additional files.

### Western blotting

Left ventricle homogenates were prepared from frozen human heart samples. Samples were homogenized in RIPA buffer (Tris 65 mmol/l NaCl 150 mmol/l, Na_2_H_2_EDTA 1 mmol/l, Triton X-100 1% v/v, Na-deoxycholate 0.5% w/v, SDS 0.1% v/v, glycerol 5% v/v, pH = 7.4) with protease and phosphatase inhibitors and protein concentration was determined with Lowry method. 15 µg of total protein per sample mixed with 2× sample buffer (Tris–HCl pH = 7.6 40 mmol/l, glycerol 20% v/v, Na_2_H_2_EDTA 1 mmol/l, Bromophenol Blue 0.01% w/v, SDS 2% w/v) was treated with 100 mM 2-mercaptoethanol at 60 °C for 20 min, separated on 10 or 12% Bis–Tris or Tris-Tricine SDS-PAGE gels and transferred onto polyvinylidene difluoride membranes (PVDF; 0.45 µm pore size; Immobilon-P, Millipore, USA). For immunodetection, the following primary antibodies were used: anti-RIP1 (1:1000, SAB3500420, Sigma-Aldrich, USA), anti-phospho-Ser^227^-RIP3 (1:1000, ab209384, Abcam, UK), anti-RIP3 (1:3000, ARP32835, Aviva Systems Biology, USA), anti-MLKL (1:750, MABC604, Millipore, USA), anti-phosphoThr^357^-MLKL (1:500, ABC234, Millipore, USA), anti-phosphoThr^358^-MLKL (1:1000, ab187091, Abcam, UK), anti-Bcl-2 (1:2000, SAB4500003, Sigma-Aldrich, USA), anti-Bax (1:1000, #2772, Cell Signaling Technology, USA), anti-PARP1 (1:1000, #9532, Cell Signaling Technology, USA), anti-PARP1p25 (1:1000, ab32064, Abcam, UK), anti-caspase-3 (1:1000, sc-98785, Santa Cruz Biotechnology, USA), anti-cleaved caspase-3 [5A1E] (1:500, #9664, Cell Signaling Technology, USA), anti-caspase-7 (1:1000, #12827, Cell Signaling Technology, USA), anti-caspase-8 (1:2000, 04-573, Millipore, USA), anti-GAPDH HRP conjugate (1:25000, G9295, Sigma-Aldrich, USA), anti-COXIV isoform 1 (1:1000, AV42784, Sigma-Aldrich, USA). Secondary antibodies used were: donkey anti-rabbit IgG-HRP (1:50000-1:100000, NA934 V, GE Healthcare Life Sciences, UK), mouse anti-rabbit light chain specific IgG-HRP (1:50000, 211-032-171, Jackson Immunoresearch, USA), goat anti-rat light chain specific IgG-HRP (1:50000, 112-035-175, Jackson Immunoresearch, USA). Signals generated with an enhanced chemiluminescence kit (Luminata Crescendo, Millipore, USA) were captured with a phosphorescence imager (myECL Imager, Thermo Scientific, USA) and quantified with myECL Image Analysis software (version 1.1, Thermo Scientific, USA). Total protein staining of membranes with Ponceau S (0.2% w/v in 3% w/v trichloroacetic acid) or Coomassie Brilliant Blue G-250 (0.25% w/v in 50% v/v isopropanol) evaluated by scanning densitometry was used as the loading control instead of housekeeping protein immunodetection [15–17]. All used chemicals were sourced from Sigma-Aldrich (USA), Alfa-Aesar (USA), SERVA (GER), CentralChem (SK) and Merck (USA).

### Statistical analysis

All data are presented in the form mean ± standard error. Group differences in measured parameters were tested with one-way ANOVA and unpaired two-tailed t test with or without Welch’s correction (based on results of F test for unequal variances). All statistical analysis was performed with GraphPad Prism version 6.00 for Windows (GraphPad Software, USA). Differences between groups were considered to be significant when P < 0.05.

## Results

### Pro-necroptotic markers in HF patients are elevated compared to controls

**Fig. 1:**
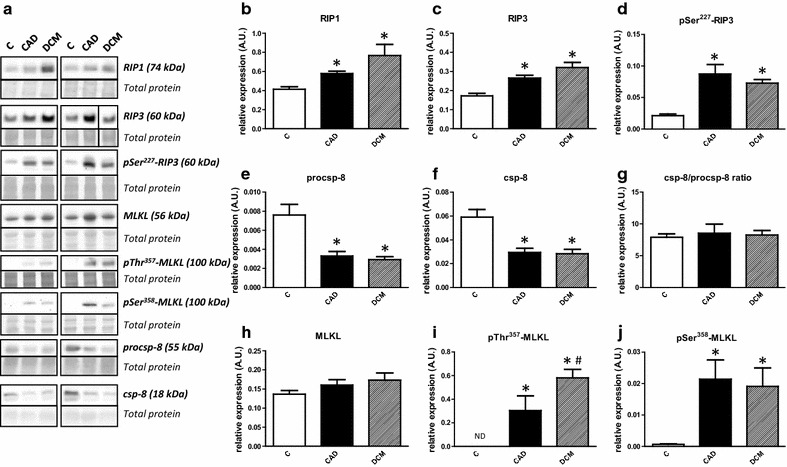
Activation of necroptotic pathway in human end-stage heart failure. **a** Representative immunoblots of RIP1, RIP3, pSer^227^-RIP3, procsp-8, csp-8, MLKL, pSer^358^-MLKL and pThr^357^-MLKL in control (C) and heart failure due to myocardial infarction (CAD) or dilated cardiomyopathy (DCM). The right RIP3 blot and its corresponding total protein stain section are spliced because of marker lane interference. **b**–**f**, **h**–**j** Quantification of RIP1, RIP3, pSer^227^–RIP3, procsp-8, csp-8, MLKL, pSer^358^-MLKL and pThr^357^-MLKL immunoblots. **g** Quantification of procsp-8/csp-8 ratio. Data are presented as mean ± SEM. *P < 0.05 vs. C; ^#^P < 0.05 vs. CAD. n = 4, 6, 10 for C, CAD and DCM respectively. *ND* non-detectable

The protein expression of some major mediators of necroptotic cell death in LVs of HF patients is shown in Fig. 1a–i. RIP1 and RIP3 expression as well its active form phosphorylated on Ser^227^ were significantly elevated in both CAD and DCM hearts, thereby indicating necroptosis induction (Fig. 1b–d). In addition, active csp-8, was severely decreased in HF samples (Fig. 1f). Interestingly though, this decrease could be solely explained through downregulation of procsp-8 expression rather than by its reduced cleavage (Fig. 1e) as csp-8/procsp-8 ratio was unchanged compared to controls (Fig. 1g). Total MLKL expression did not significantly differ among tested groups (Fig. 1h). Importantly, both key terminal markers of necroptosis, pThr^357^-MLKL and pSer^358^-MLKL, were present in all HF samples while they were expressed at almost undetectable levels in controls (Fig. 1i, j). Moreover, pThr^357^-MLKL expression, unlike pSer^358^-MLKL was significantly higher in DCM hearts than CAD ones. However, the molecular weight of the observed band was 100-110 kDa suggesting dimer formation. As MLKL oligomerization post phosphorylation should involve trimer, tetramer or hexamer formation with possible translocation into cellular membranes [7], antibody non-specificity was also suspected even though the observed pattern was concordant with our hypothesis. Therefore, in order to support these findings, we performed a subcellular fractionation with Triton X-114 (Additional file 1: Figure S1A–G). Due to limited amount of human heart tissue, the analysis was performed with pooled samples only and thus differences among groups could not be analyzed statistically. As shown in Figure S1A, pThr^357^-MLKL signal was detected ~100 kDa on top of MLKL (Additional file 1: Figure S1B,C) in CAD and DCM samples but not in controls. These findings are consistent with those from whole tissue lysates. Of note, the signal was present in aqueous and insoluble fractions. Whether this signal represents a dimer or some post-translationally modified form of MLKL remains to be determined. Unlike pThr^357^-MLKL, RIP3 was detected in all tested fractions with notable increases in cytoplasm and membrane compartments of HF samples as would be expected for active necroptosis (Additional file 1: Figure S1D). No discernible pattern of distribution could be seen for RIP1 (Additional file 1: Figure S1E).

### Lack of apoptosis in end-stage heart failure

**Fig. 2:**
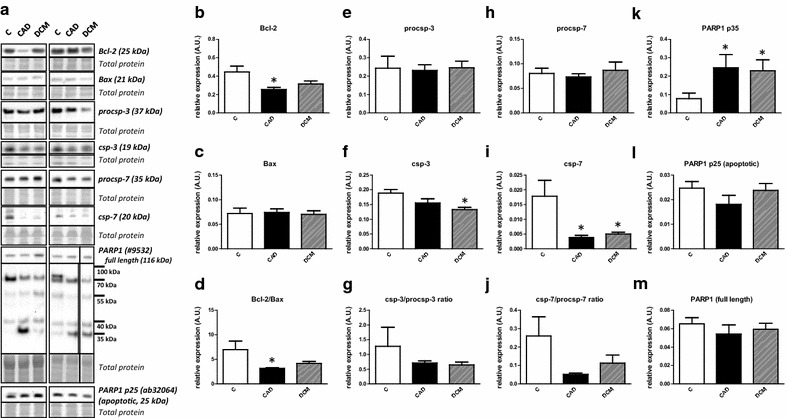
Lack of significant changes in the expression of apoptotic markers in human end-stage heart failure. **a** Representative immunoblots of Bcl-2, Bax, procsp-3, csp-3 and PARP1 in control (C) and heart failure samples due to myocardial infarction (CAD) and dilated cardiomyopathy (DCM). The right PARP1 blot and its corresponding total protein stain section are spliced because of marker lane interference. **b**, **c**, **e**, **f**, **h**–**m** Quantification of Bcl-2, Bax, procsp-3, csp-3, procsp-7, csp-7, PARP1 p35, PARP1 p25 and total PARP1 immunoblots. **d**, **g** Quantification of Bcl-2/Bax, csp-3/procsp-3 and csp-7/procsp-7 ratios. Data are presented as mean ± SEM. *P < 0.05 vs. C. n = 4, 6, 10 for C, CAD and DCM respectively

Expression of apoptotic markers is summarized in Fig. 2a–m. Bcl-2 levels were lower in both HF groups; however, only the comparison of C vs. CAD reached significance (Fig. 2b). Bax was found to be essentially unchanged in both HF groups (Fig. 2c). These changes were reflected in the Bcl-2/Bax ratio (Fig. 2d). Caspase-3 (csp-3) displayed a significant decrease in DCM compared to controls (Fig. 2f) while this effect was lost when csp-3/procsp-3 ratio was quantified (Fig. 2g). Active caspase-7 (csp-7) expression was significantly downregulated in all failing hearts irrespective of the etiology while procsp-7 and csp-7/procsp-7 did not differ among the groups (Fig. 2h–j). In addition to these apoptotic markers, we analyzed PARP1, one of the principal targets of active csp-3 and csp-7 [18]. Total PARP1 was comparable in all groups (Fig. 2m). However, p89 fragment with reduced DNA binding capacity, a sensitive marker for apoptosis [19], could not be detected in any of the samples with the antibody used while its counterpart apoptotic DNA repair enzyme inhibiting p25 fragment (N terminal peptide) did not differ in failing hearts (Fig. 2l). Other fragments of PARP1 with uncertain identity could be identified at ~70, ~55, ~40 and ~35 kDa. The expression of the first 3 fragments did not differ among the groups. On the other hand, the 35 kDa fragment, which is associated with µ-calpain activity [18], was significantly upregulated in both HF groups (Fig. 2k).

## Discussion

In this study, we have indicated for the first time that (i) left ventricular samples of human HF are characterized by elevated expression of major factors of necroptosis induction, (ii) DCM is characterized by a higher expression of a main executive necroptotic protein—pThr^357^-MLKLas compared to CAD and (iii) that these main proteins of necroptotic cell death outweigh the apoptotic pathway.

Both HF groups showed upregulation of RIP1 and RIP3. However, as these protein kinases (mainly RIP1) exhibit constitutive activity [6] their upregulation does not definitively indicate necroptosis. Therefore, active pronecroptotic forms of proteins of RIP1-RIP3 axis, should be presented to support findings about the presence of necroptosis in diseased tissue. To follow this approach, we analyzed tissue expression of pSer^227^-RIP3, a useful necroptotic marker which has been suggested to be present during active necroptosis [20]. pSer^227^-RIP3 was highly elevated in all failing hearts irrespective of the etiology suggesting that RIP3 and its phosphorylation could be involved in human HF necroptosis. Indeed, a critical role of RIP3 in the myocardial necroptotic cascade has been demonstrated in the work of Luedde et al. [12] and further supported by another study [13]. In fact, mice deficient in RIP3 have shown significantly improved ejection fraction, less hypertrophy and inflammatory response after permanent coronary artery ligation. Furthermore, RIP3 activation has been indicated to be essential for activation of CaMKII with subsequent mPTP opening and induction of cell death of necrotic phenotype. It can be mentioned that this axis of RIP3-dependent necroptosis was found in both I/R and doxorubicin-induced cardiotoxicity [13], animal model analogues to types of heart failure referred to in our study. The involvement of this protein kinase, a key regulator of Ca^2+^ homeostasis in cardiomyocytes in myocardial I/R-induced necroptosis, has also been proposed in our recent study showing that the inhibition of CaMKII reversed certain changes in pronecroptotic markers what was accompanied by improved contractile function [10]. It should be noted, however, that the pronecroptotic axis involving RIP3-CaMKII resulting in cell death via effects on mitochondria [13] is contradictory to studies showing that necroptosis occurs due to plasma membrane disruption as a consequence of recruitment of MLKL following RIP1-RIP3 activation [7, 12, 20].

Here, we have also found that the expression of csp-8, a negative regulator of necroptosis, was significantly downregulated in end-stage HF. Furthermore, we have investigated a specific molecular marker of necroptosis execution which until very recently was impossible to probe for. However, this fact changed with MLKL phosphorylation at Thr^357^ or Ser^358^ being described as a crucial terminal marker of necroptosis [7, 21, 22]. Only one of these residues has been shown to be essential for necroptosis execution [23]. In fact, this post-translationally modified form of MLKL has been found to oligomerize (however, the number of monomers required for oligomer assembly is still a subject of investigation), translocate into the cellular membrane and thereby alter the influx of ions, mainly Na^+^ and Ca^2+,^ or increase non-specific membrane permeability [7, 21, 22]. Thus, disruption in ion homeostasis leading in cell oncosis has been proposed to underlie the cytotoxic action of phospho-MLKL. In this study, these cytotoxic forms of MLKL phosphorylated at Thr^357^ and Ser^358^ were detectable in all HF samples supporting the implication of RIP1, RIP3 and p-Ser^227^-RIP3 expression data and the hypothesis that this cell death mode might be involved in the pathogenesis of HF [11]. These results are further supported by data obtained from Triton X-114 fractionation which has revealed the presence of this critical necroptotic marker in phases representing the cytoplasm, nuclei and possibly lipid rafts [24]. This may indicate that the execution of necroptosis in end-stage HF is not restricted to only cytoplasm and membranes but also may involve the nucleus. The suggested nuclear translocation is in agreement with very recent findings reported from the colorectal cell line HT29 [25].

According to available knowledge about the regulation of programmed cell death types, when necroptosis is not being activated the pathways default to apoptotic processes usually by the means of csp-8 [6]. Indeed, the inhibition or depletion of this caspase has been shown to inhibit apoptosis while promoting necroptosis and, analogously, active csp-8 leads to cleavage of RIP1 and RIP3 which subsequently promotes apoptosis and prevents necroptosis [26]. Since csp-8 was found to be significantly downregulated, our data support the statement that apoptosis is unlikely to be significantly increased in human end-stage HF [2, 27, 28] and that necroptosis, rather than apoptosis itself, might be critical for the determination of a number of viable cells in HF [14]. It can be mentioned that the observed downregulation of csp-8 seems to be an effect attributable to reduced procsp–8 expression and not its cleavage. While we did not seek an explanation for this novel csp-8 related finding, a plausible link between procsp–8 downregulation, necroptosis and miR-874 has been recently described in cardiomyocytes [29]. In line with the theoretical paradigm and our hypothesis about increased necroptosis at the expense of apoptosis in HF, we have shown that protein expression of main apoptotic markers was either mostly unchanged or reduced. Indeed, the levels of Bax, a proapoptotic protein, were unchanged while expression of Bcl-2, counterbalancing the effects of Bax, was decreased or unchanged in failing hearts. Importantly, increased PARP1 apoptotic fragmentation does not seem to occur in these diseased hearts. In fact, an 89 kDa fragment (C-terminal) with reduced DNA binding capacity released into the cytosol from the nucleus after cleavage could not be detected [30]. The second part of PARP1 produced during apoptotic processing, a 25 kDa fragment known to be retained in the nucleus where it inhibits the active DNA repairing PARP1 [31], could be detected but did not differ among the samples. This cleavage of PARP1 producing 25 and 89 kDa fragments is mediated by executioner caspases csp-3 and csp-7 [18] expression of which was unchanged or decreased depending on the type of heart failure. Thus, these data referring to apoptotic pathway indicate that this particular regulated cell death does not play a major role in pathogenesis of HF. Studies of others [2–5, 27, 32, 33] are in line with our report arguing against the importance of apoptosis in HF. On the other hand, it should be also noticed that some studies present the argument that even a small percentage of apoptotic cardiac myocytes is sufficient to induce heart failure [32, 33].

## Conclusions

**Fig. 3:**
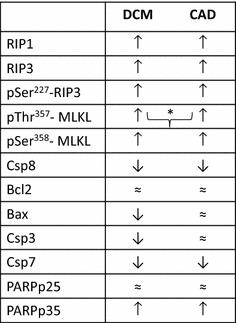
Schematic illustration of main findings indicating the levels of necroptotic and apoptotic proteins. Symbols ↑, ≈ , ↓ indicate the expression of particular proteins compared to their levels in healthy non-failing hearts. Symbol] is used to indicate comparison between failing groups. Thus, symbol] * shows the higher expression of the particular protein in DCM (dilated cardiomyopathy) compared to CAD (ischemic cardiomyopathy)

Our results provide the first evidence of either increased expression or selective presence of necroptotic proteins in human end-stage HF, which indicate both necroptosis activation and execution. Although both of the active cytotoxic phosphorylated forms of MLKL were upregulated in failing hearts irrespective of the etiology, pThr^357^-MLKL levels were higher in DCM than in CAD hearts. Complex analysis of major apoptotic proteins has not revealed a defining role of apoptotic cell death in the pathomechanisms of this disease (Fig. 3). By taking into consideration the fact that necroptosis, like any cell death, limits the number of functional cardiomyocytes, it can be assumed that these changes might also underlie the depressed function of failing hearts. In addition, it can also be suggested that interfering with RIP1-pSer^227^-RIP3-phospho-MLKL signaling might be a pharmacological intervention worth pursuing to prevent or retard the progression of HF.

## Limitations

The main limitation of the study is that our conclusions are only based on protein data. However, our goal was to examine if there are differences in necroptotic proteins depending on the etiology of heart failure, what is a completely new view onto the pathology of the disease. Likewise, patients with HF were treated with several drugs (beta-blockers, angiotensin-converting enzyme inhibitors, diuretics, etc.), while healthy control subjects were not given this chronic medication. In the human studies like this, this factor cannot be selectively excluded.

## Additional file

**Additional file 1.** Additional data on patient characteristics and Triton X-114 fractionation.

## Abbreviations

Bax: Bcl-2 associated protein X
Bcl-2: B-cell leukemia/lymphoma 2
Csp-3: caspase-3
Csp-7: caspase-7
Csp-8: caspase-8
HF: heart failure
LV: left ventricle
MLKL: mixed-lineage kinase domain-like
NYHA: New York Heart Association
PARP1: poly (ADP-ribose) polymerase 1
RIP1: receptor-interacting protein 1
RIP3: receptor-interacting protein 3
RIPA: radioimmunoprecipitation assay
