# Erratum to: Analysis of necroptotic proteins in failing human hearts

**DOI:** 10.1186/s12967-017-1207-7

**Published:** 2017-05-11

**Authors:** Adrián Szobi, Eva Gonçalvesová, Zoltán V. Varga, Przemyslaw Leszek, Mariusz Kuśmierczyk, Michal Hulman, Ján Kyselovič, Péter Ferdinandy, Adriana Adameová

**Affiliations:** 1 Department of Pharmacology & Toxicology, Faculty of Pharmacy, Comenius University in Bratislava, Odbojárov 10, 832 32 Bratislava, Slovakia; 2 Department of Heart Failure & Transplantation, The National Institute of Cardiovascular Diseases, Bratislava, Slovakia; 3 Department of Pharmacology & Pharmacotherapy, Semmelweis University, Budapest, Hungary; 4 Institute of Cardiology, Warsaw, Poland; 5 Clinic of Heart Surgery, The National Institute of Cardiovascular Diseases, Bratislava, Slovakia

## Erratum to: J Transl Med (2017) 15:86 DOI 10.1186/s12967-017-1189-5

In the original version of this article [1], published on 28 April 2017, the name of author ‘Zoltán V. Varga’ was wrongly displayed.

Originally the author name has been published as:

- Zoltán Varga

The correct author name is:

- Zoltán V. Varga

The original publication of this article has been corrected.
