# Supplementary material for: Analysis of necroptotic proteins in failing human hearts
Source: J Transl Med. 2017 Apr 28;15:86. doi: 10.1186/s12967-017-1189-5 (PMC5410070; doi:10.1186/s12967-017-1189-5)
Supplement: Supplementary file 1 — Additional file 1. Additional data on patient characteristics and Triton X-114 fractionation. [file 12967_2017_1189_MOESM1_ESM.docx]

**SUPPLEMENTAL MATERIAL**

**for**

**Analysis of necroptotic proteins in failing human hearts**

**EXPANDED METHODS**

**Triton X-114-based subcellular fractionation by phase separation**

A modified Triton X-114 (TX114) temperature-induced crude fractionation of proteins into an aqueous phase (Aq), a detergent phase (Dg) and an insoluble pellet (In) was used. After separation, Aq contains soluble cytosolic proteins and Dg phase is enriched in membrane and membrane-associated proteins. In phase contains cytoskeletal proteins, nuclei and some damaged organelles but also so called detergent-resistant membrane domains which are not amenable to extraction by TritonX-114 [1, 2]. Therefore the In phase likely contains a certain fraction of membrane proteins, such as proteins of lipid rafts, which is a limitation of this method. Per-group pooled samples of LVs (14-17 mg of tissue per sample) were crushed in liquid nitrogen into a fine-powder to which 2% TX114 buffer (750 µl/10 mg; TX114 2% v/v, HEPES 150 mmol/l, NaCl 20 mmol/l, 5% glycerol v/v) with protease and phosphatase inhibitors was added. The mixture was powdered again, transferred to a chilled eppendorf tube and vortexted for 15 min. Homogenates were passed through a 1 ml pipette tip 10-15 times and then centrifuged at 14000 g for 10 min at 4 °C. The TX114-containing supernatant was transferred to another pre-chilled tube while the pellet was washed with 2% TX114 buffer, resuspended in fresh 2% TX114 buffer and recentrifuged at 14000 g for 10 min at 4 °C. The cleaned pellet without supernatant was put into a ‑80 °C freezer until further processing. Phase separation of the TX114-containing supernatant was induced by submerging the tube into 25-27 °C water for 5 min. Afterwards, it was centrifuged at 900 g for 5 min at 25 °C. The lighter Aq phase was transferred to another tube while the Dg phase enriched in membrane proteins was left in the original one. Washing of the Aq phase was performed by adding 10% TX114 buffer (20% of Aq volume), quickly chilling it in liquid nitrogen (for the 2 phases to combine), incubating in 25-27 °C water for 5 min and centrifuging again at 900 g for 5 min at 25 °C. The Dg phase was washed in the same manner but base HEPES buffer (HEPES 150 mmol/l, NaCl 20 mmol/l, 5% glycerol) was used instead. Unwanted phases were subsequently removed and the washing procedure was repeated 2 more times. Finally, the volume of the Dg phase was adjusted to that of the Aq phase with base HEPES buffer and 20% w/v SDS solution was added to both phases to a final SDS concentration of 1.5%. The TX114 insoluble pellet stored earlier was thawed and dissolved in a volume of RIPA buffer (the same as used in Western blotting) equal to the volume of final Aq and Dg homogenates. RIPA-insoluble material was discarded after a 10 min 14000 g centrifugation at 4 °C. Purity of isolated fractions was checked by immunoblotting for cytosolic GAPDH (Figure S1F) and membrane COXIV|1 (Figure S1G). GAPDH was present only in the aqueous fraction while COXIV|1, a predominantly mitochondrial membrane protein, was in the detergent fraction and to a lesser degree in the pellet fraction as can be expected with this method. Due to low protein concentration detergent fractions were concentrated with acetone precipitation to improve signal detection during Western blotting.

**Acetone precipitation of detergent phase proteins**

120 µg of detergent phase proteins (~200 µl; as determined with Lowry assay) obtained with TX114-based fractionation were transferred to a 2 ml tube and mixed with 150 µl of 4 mol/l NaCl solution to promote protein precipitation [3]. After adding 4 volumes of -20 °C acetone, proteins were precipitated overnight at -20 °C. The tubes were centrifuged at 7500 g for 5 min at 4 °C, the supernatants were discarded and the resulting pellets were dissolved in 65 µl of a 1:1 mixture of RIPA and 2x sample buffer. Prepared samples (~6-times more concentrated than input) were then reduced with 2-mercaptoethanol for use in SDS/PAGE and Western blotting.

**Western blot image blending**

pThr^357^-MLKL/MLKL blended image was obtained as follows. pThr^357^-MLKL blot was developed, then it was stripped, reincubated with its respective secondary antibody to check for residual primary antibody, re-stripped and finally probed with an anti-MLKL antibody from a different host. Molecular weight markers visible on membranes were used to properly align the two blots with respect to their position and rotation. GIMP software (ver 2.8.10) tool “Color to Alpha” was used to turn the black background transparent and next “Colorize” was used to turn them different colors. The images were then loaded in Inkscape (ver 0.48.4) where a custom multi-effect filter (background image-MLKL, source image-pThr^357^-MLKL) was applied to the two images. Finally, the background was set to black to obtain the final image.

**
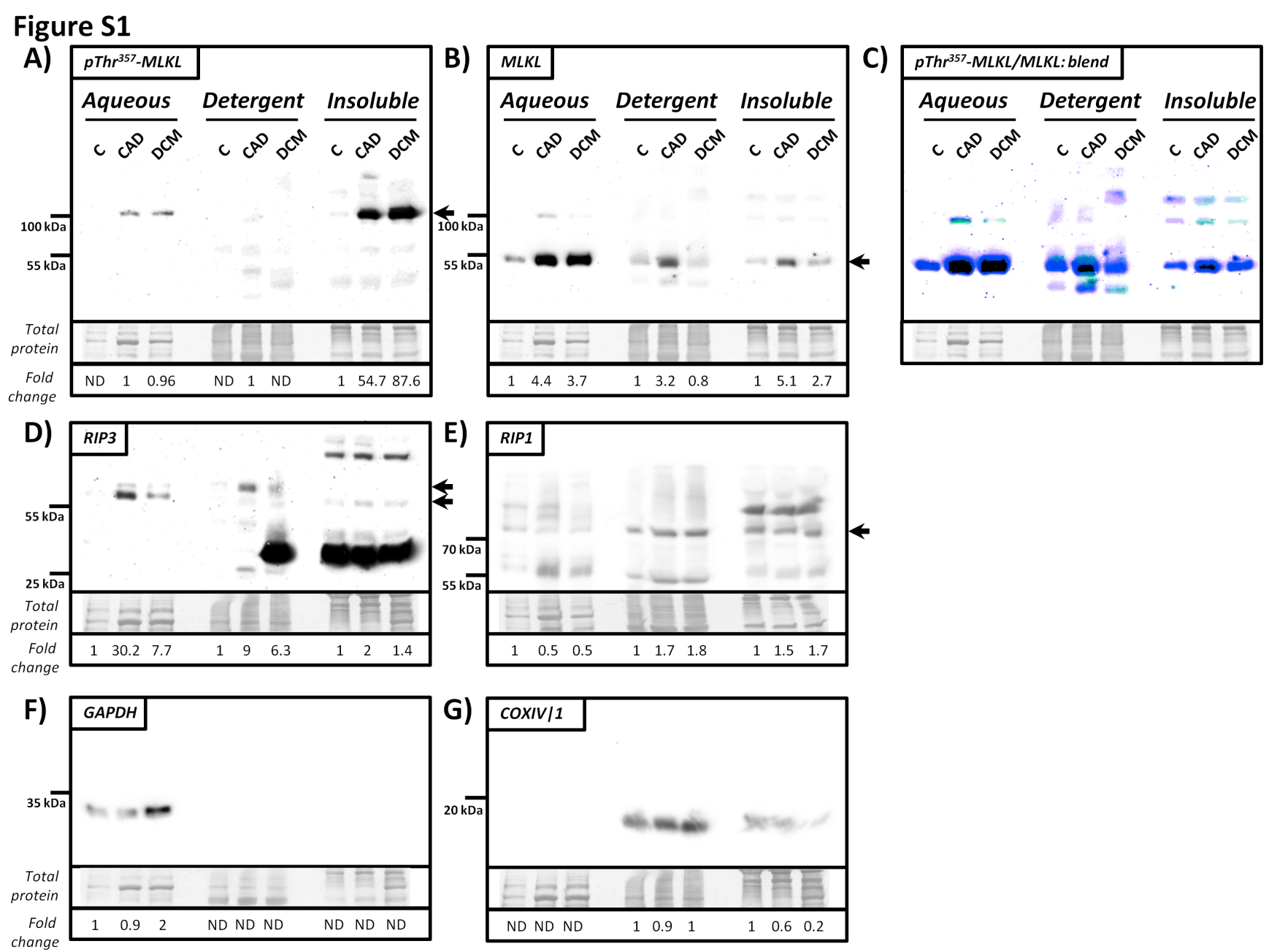
ADDITIONAL FIGURES AND TABLES**

**Figure S1**

Subcelullar fractionation of LV homogenates using TX-114 in pooled samples (n=4, 6, 10 / pooled samples for C, CAD and DCM; respectively). Fractionated and SDS-PAGE separated samples were probed for **a)** pThr^357^-MLKL followed by stripping and **b)** reprobing for total MLKL. **c)** Blended image of **a)** and **b)** clearly showing a perfect overlap (turquoise) between pThr^357^-MLKL and total MLKL (blue-black) signals ~100 kDa. **d)** RIP3 immunoblot of fractionated samples. **e)** RIP1 immunoblot of fractionated samples. **f-g)** Immunoblots of GAPDH and COXIV|1 respectively demonstrating efficient fractionation by TX114 as well as presence of non-nuclear membrane proteins in the pellet fraction. C – control, CAD – HF due to myocardial infarction, DCM – HF due to dilated cardiomyopathy, ND – not detectable. In blots with multiple bands, arrows denote particular bands used to calculate relative differences in protein expression normalized to protein expression of controls. RIP3 and pThr^357^-MLKL blots were gamma-enhanced for visual clarity.

| **Table S1**  **Clinical, echocardiographic, hemodynamic and biochemical characteristics of patients and control healthy donors** | | | |  |
| --- | --- | --- | --- | --- |
|  | Control | CAD | DCM |  |
|  |  |  |  |  |
| Number of samples | 4 | 6 | 10 |  |
| Gender (male/female) | 2/2 | 5/1 | 8/2 |  |
| Age (years) | 30 ± 5 | 52 ± 4* | 47 ± 3* |  |
|  | |  |  |  |
| LVEF (%) | n.a. | 21 ± 1 | 18 ± 1 |  |
| LVEDD (mm) | n.a. | 75 ± 4 | 72 ± 3 |  |
| RVEDD (mm) | n.a. | 34 ± 3 | 35 ± 1 |  |
| PAP sys (mm Hg) | n.a. | 44 ± 7 | 53 ± 5 |  |
| PAP dia (mm Hg) | n.a. | 21 ± 4 | 27 ± 3 |  |
| PCWP (mmHg) | n.a. | 18.8 ± 3.7 | 25.9 ± 1.9 |  |
| PVR (mmHg.min/l) | n.a. | 2.6± 0.3 | 3.1± 0.5 |  |
| CI (l/min/m^2^) | n.a. | 2.2 ± 0.1 | 1.8 ± 0.1 |  |
| QT (s) | n.a. | 0.42 ± 0.01 | 0.41 ± 0.02 |  |
|  |  |  |  |  |
| NT-proBNP (ng/l) | n.a. | 3541 ± 1035 | 6571 ± 1682^†^ |  |

**Table S1**

The two end-stage HF groups of ischemic and non-ischemic origin did not significantly differ in echocardiographic or hemodynamic parameters. Elevated NT-proBNP levels were observed in patients with dilated cardiomyopathy compared to patients with failing hearts due to myocardial infarction. LVEF – left ventricular ejection fraction; LVEDD – left ventricular end-diastolic diamter; RVEDD – right ventricular end-diastolic diamter; PAP sys – systolic pulmonary artery pressure; PAP dia – diastolic pulmonary artery pressure; PCWP – pulmonary capillary wedge pressure; PVR – pulmonary vascular resistance; CI – cardiac index; QT – QT interval; NT-proBNP – N-terminal prohormone of brain natriuretic peptide; Data are presented as mean ± SEM. * P<0.05 vs. C; ^†^ P<0.05 vs. CAD.

**ADDITIONAL REFERENCES**

1. Bordier C. Phase separation of integral membrane proteins in Triton X-114 solution. J Biol Chem. 1981;256:1604-7.
2. Brown DA. Lipid rafts, detergent-resistant membranes, and raft targeting signals. Physiology (Bethesda). 2006;21:430-9.
3. Crowell AM, Wall MJ, Doucette AA. Maximizing recovery of water-soluble proteins through acetone precipitation. Anal Chim Acta. 2013;796:48-54.
